# A Multiplex Thyroid-Specific Assay for Quantification of Circulating Thyroid Cell-Free RNA in Plasma of Thyroid Cancer Patients

**DOI:** 10.3389/fgene.2021.721832

**Published:** 2021-08-25

**Authors:** Samantha Peiling Yang, Lian Chye Winston Koh, Kiat Whye Kong, Rajeev Parameswaran, Kelvin Siu Hoong Loke, Kee Yuan Ngiam, Wee Boon Tan, Thomas Loh, David Chee Eng Ng, Boon Cher Goh, Joanne Ngeow, E. Shyong Tai

**Affiliations:** ^1^Endocrinology Division, Department of Medicine, National University Hospital, Singapore, Singapore; ^2^Endocrinology Division, Department of Medicine, Yong Loo Lin School of Medicine, National University of Singapore, Singapore, Singapore; ^3^Molecular Engineering Lab, Institute of Molecular and Cell Biology (IMCB), A∗STAR, Singapore, Singapore; ^4^Department of Endocrine Surgery, National University Hospital, Singapore, Singapore; ^5^Department of Nuclear Medicine and Molecular Imaging, Singapore General Hospital, Singapore, Singapore; ^6^Department of Otolaryngology Surgery, National University Hospital, Singapore, Singapore; ^7^Department of Medical Oncology, National University Hospital, Singapore, Singapore; ^8^Cancer Genetics Service, Division of Medical Oncology, National Cancer Centre, Singapore, Singapore; ^9^Lee Kong Chian School of Medicine, Nanyang Technological University, Singapore, Singapore

**Keywords:** thyroid-specific transcripts, thyroglobulin antibody positivity, thyroid cancer surveillance follow-up, thyroglobulin assay, thyroid cancer recurrence

## Abstract

**Background:**

The standard of care for thyroid cancer management is thyroidectomy and adjuvant radioactive iodine (RAI). There is a paucity of clinical tool that quantifies residual thyroid volume reliably for precise adjuvant RAI dosing. Serum thyroglobulin (TG), tumour marker for thyroid cancer, takes 4 weeks for complete clearance due to its long half-life, and might be undetectable in 12% of structural disease patients. It detects recurrence with a sensitivity of 19–40%, mainly attributed to issue of TG antibody interference with TG immunometric assay. We hypothesise that the quantity of thyroid-specific cell-free RNA (cfRNA) is indicative of amount of thyroid tissues, and that during thyroid surgery, cfRNA levels decrease accordingly.

**Methods:**

We identified 11 biologically significant and highly expressed thyroid-specific targets from Human Protein Atlas and literature. To assess for a fall in thyroid-specific cfRNA level, we recruited 16 patients undergoing thyroid surgery or RAI for malignant or benign thyroid disease, and tracked longitudinal trend of cfRNA. To assess the utility of cfRNA in detecting metastatic thyroid cancer, cfRNA of 11 patients at intermediate to high risk of recurrence was measured during surveillance and at time of clinical recurrence.

**Results:**

The multiplex assay was capable of amplifying and quantifying multiple thyroid-specific genes in a single reaction. The selected targets were amplified successfully from RNA extracted directly from the thyroid (positive control), indicating that they were highly expressed within thyroid tissue. These cfRNAs were present in plasma, in amounts quantifiable using qRT-PCR. Four cfRNA transcripts (TPO, GFRA2, IVD, TG) fell post-treatment in more than 50% of cohort. The thyroid peroxidase (TPO) cfRNA fell post-therapy in 63% of cohort by 80%, as early as 1 day post-treatment, supporting the potential role as early indicator of remnant thyroid tissue volume. We demonstrated the clinical relevance of circulating TPO cfRNA by tracking temporal changes in setting of peri-treatment, recurrence, and TG Ab positive state.

**Conclusion:**

Using a multiplex pre-amplification approach, the TPO cfRNA was a potential biomarker that can track residual thyroid mass. It can be further optimised for quantification of thyroid volume to guide RAI doses and for detection of thyroid cancer recurrence.

## Introduction

The current standard of care for management of thyroid cancer has been total thyroidectomy followed by post-operative adjuvant radioactive iodine (RAI) for majority of patients. The decision for RAI and the I–131 dosage, ranging from 30 to 250 mCi per dose, is determined by the predicted tumour burden and risk of recurrence. In patients with advanced distant metastatic disease, higher doses of 100–250 mCi of RAI is usually recommended (Haugen et al., 2016). There is a lack of precision for estimation of adjuvant RAI treatment dosages due to the absence of a clinical tool that quantifies residual thyroid tissue volume reliably. The current tumour marker, serum thyroglobulin that has a half-life of 65 h, may take at least 7–10 half-lives (4 weeks) for complete thyroglobulin clearance in the absence of metastases (Hocevar et al., 1997). One study had shown that undetectable baseline thyroglobulin at 1 month post-surgery was associated with absence of structural disease on post-operative scans (Giovanella et al., 2008), whereas another study showed that undetectable thyroglobulin levels 2–6 months post-surgery was associated with radioactive iodine avid metastatic disease in 12% of cases (Robenshtok et al., 2013).

The role of post-surgical pre-ablative diagnostic radioiodine scan to estimate the remnant amount of thyroid tissue had been explored. However, there had been studies reporting that low doses of RAI given for these diagnosis was associated with higher risk of remnant ablation failure, hypothesised to be due to the stunning effect of the RAI dose for diagnostic scan rendering lower RAI uptake with the subsequent treatment dose of RAI (Hu et al., 2004; Verburg et al., 2009). As such, this is not routinely done in clinical practice (Haugen et al., 2016).

Using circulating tumour DNA (ctDNA) to estimate residual thyroid cancer tissue can be challenging. In a study evaluating the performance of ctDNA using digital PCR, the authors pointed out that ctDNA was detected mainly in patients with solid tumours outside the brain (112 of 136; 82%). In contrast, less than 50% of patients with medulloblastomas or metastatic cancers of the kidney, prostate, and thyroid harboured detectable ctDNA (Bettegowda et al., 2014). Compared to ctDNA with only two copies per cell, multiple copies of tissue-specific RNA are present, providing a higher chance of detection. As such, cfRNA can potentially overcome the challenges of using small input amount of plasma and the need for higher sensitivity for detection of thyroid tissues.

Unlike other solid organ malignancies where the primary organ is still *in situ* after excision of tumour, thyroid cancer treatment involves removal of the entire thyroid gland as part of the treatment strategy. This would have led to the fall of thyroid-specific cell-free RNA (cfRNA) transcripts. Therefore any elevation in these circulating tissue-specific RNA levels could potentially indicate significant residual thyroid tissue or tumour burden since these transcripts reflect the presence of the cells originating from thyroid tissue. We had previously shown that cfRNA can change over 6–8 h (Koh et al., 2014). As such, cfRNA could be a potential real-time indicator of the residual thyroid tissue volume as opposed to tumour marker, serum thyroglobulin that may take at least 4 weeks for complete thyroglobulin clearance in the absence of metastases.

In our study, we aim to demonstrate the feasibility of cfRNA in tracking thyroid tissue volume as a proof-of-concept to allow for further clinical study design to (1) examine the clinical utility in thyroid cancer risk stratification for guidance on RAI treatment, and (2) to assess its performance in detecting thyroid cancer recurrence.

Thyroid cancer surveillance for recurrence is performed using ultrasound scan of neck, cross-sectional imaging in some patients, along with serum thyroglobulin (TG) to monitor disease burden in response to treatment (Robbins et al., 2004). It detects recurrence in thyroid cancer with a sensitivity of 19–40% and specificity of 92–97% (Schlumberger et al., 2007). The rather low sensitivity of thyroglobulin for detecting recurrence leaves room for the development of molecular tools. In addition, cfRNA will address the issue of anti-thyroglobulin antibodies (present in 25% of thyroid cancer patients), that affects the reliability of thyroglobulin immunoassay (Giovanella et al., 2011; Spencer, 2011). Similarly, in a review of papillary thyroid carcinoma cohort in our institution, 24% of the patients (20/83) had anti-thyroglobulin antibodies (TG Ab) (Sek et al., 2021). TG Ab interferes with the serum thyroglobulin immunometric assay (IMA) measurement causing TG underestimation with the risk of missing detection of persistent or recurrent thyroid cancer. In this clinical setting, alternative methods of assessing serum TG using radioimmunoassay (RIA) or liquid chromatography/tandem mass spectrometry (LC-MS/MS) had been studied (Netzel et al., 2015). These were reported to have no interference from TG Ab. However, the functional sensitivity of RIA (0.5–1.0 ug/L) and LC-MS/MS (1–2 ug/L) are lower than IMA method (0.05–1.0 ug/L), and they are not readily available at most institutions. In this clinical scenario, TG Ab had been used as a surrogate tumour marker for thyroid cancer. However, TG Ab reduction in level post-treatment is usually delayed (half-life 10 weeks). The TG Ab titre falls in 75% of patients following complete treatment, but only 50% of these patients have undetectable TG Ab after 4 years of follow-up. It is uncertain if this TG Ab persistence is due to continued TG production by persistent thyroid tissues not detected by imaging or a stigma of continued activity of plasma cells (Spencer, 2013). Lastly, patients with poorly differentiated thyroid cancers lose the ability to produce thyroglobulin, making the measurement of thyroglobulin an unreliable reflection of tumour burden in these patients (Robbins et al., 2004).

Targeting circulating RNA of thyroid origin in thyroid cancer patients is a promising biomarker to monitor disease status. Utility of thyroid-specific mRNA transcripts such as thyroid peroxidase (TPO), sodium-iodide symporter (NIS), thyroglobulin (TG), and thyroid stimulating hormone receptor (TSHR) in predicting thyroid cancer recurrences and metastases had been assessed previously using whole blood (Barzon et al., 2004). TPO and TSHR mRNA extracted from whole blood showed significant correlation with disease status; they showed higher specificity (65–81%) but lower sensitivity (40–53%), while TG and NIS mRNA showed high sensitivity (60–73%) but low specificity (29–48%). Thyroid-specific cell free RNA (cfRNA) from the plasma fraction instead of whole blood can provide better sensitivity without the cellular background and will have to be studied to ascertain if it could offer better sensitivity than TG for use in detection of cancer recurrence. cfRNA had been used for tumour surveillance in other tumours; for example, cfRNA showing PD-L1 expression had been monitored in metastatic gastric cancer patients (Shen et al., 2016).

## Materials and Methods

### Clinical Sample Collection

In order to demonstrate a fall of thyroid-specific cfRNA after thyroid surgery, 16 patients undergoing total or hemi-thyroidectomy for thyroid cancer or benign thyroid disease were recruited. Peripheral blood pre- and post-surgery was assessed for a fall in levels of circulating thyroid-specific cfRNA. To establish the temporal trend of the cfRNA titre with thyroidectomy, post-surgical cfRNA was evaluated at multiple time points at the following time intervals 24 h, 1 week, 1 month, and 6 months post-surgery. There were two healthy volunteers included as control group.

To assess the utility of thyroid-specific cfRNA in detecting recurrent or persistent metastatic thyroid cancer, peripheral blood of 11 patients at American Thyroid Association (ATA)- intermediate to high of recurrence (Haugen et al., 2016) was collected during surveillance visits and at time of clinical recurrence. The cfRNA titres was correlated with serum TG, thyroid uptake on radioiodine scan (if available), and neck ultrasound.

Treatment response was classified as excellent, indeterminate, biochemical incomplete or structural incomplete based on the American Thyroid Association (ATA) thyroid cancer management guidelines (Haugen et al., 2016). Patients were considered to have an excellent treatment response if they had non-stimulated TG < 0.2 ug/L or stimulated serum TG < 1.0 ug/L after RAI ablation, with no detectable TG Ab and no structural disease on neck ultrasound scan and/or cross-sectional or radioiodine imaging. Patients were considered to have an indeterminate response if they had a non-stimulated serum TG between 0.2 and 1.0 ug/L, stimulated serum TG ≥ 1–10 ug/L after RAI ablation, stable or declining TG Ab or non-specific changes on neck ultrasound scan and/or cross-sectional or radioiodine imaging. Patients were considered to have biochemical incomplete treatment response if they had a non-stimulated TG > 1 ug/L after RAI ablation, simulated serum TG > 10 ug/L or rising TG/TG Ab values without structural evidence of disease on imaging. Patients with structural evidence of disease on imaging were considered to have structural incomplete response.

The study was conducted from May 2018 to Oct 2020, and the study protocol was approved by the local ethics board committee (NHG DSRB Study Reference Number: 2017/00632). Written informed consent was obtained from all participants.

### Clinical Laboratory Measurement

The serum TG was measured using immunometric assay, e411 (Roche) or E170 (Roche) with a functional sensitivity of 0.5 ug/L before 2015. Subsequently, the TG assay was changed in 2015 to Kryptor (Brahms) with a functional sensitivity of 0.15 ug/L, and to e411 (Roche) in 2017 with a functional sensitivity of 0.1 ug/L. Anti-TG antibodies were measured using radioimmunoassay Immulite 2000 (Siemens) assay before 2017, or e411 (Roche) from 2017.

The analytical functional sensitivity of TG Ab was 20 IU/mL for Immulite 2000 (Siemens) assay and Roche assay. We considered a detectable level of TG Ab according to the functional sensitivity (limit of quantification) as TG Ab positive state. Of note, reference values of TG Ab are reported to distinguish individuals with and without thyroid autoimmune disease. Whereas detectable concentrations of TG Ab (i.e., above the functional sensitivity) even below the normal reference range cut-off can interfere with serum TG (Spencer et al., 2014).

### Selection of Thyroid-Specific Targets for Measurements in Plasma

A data-driven approach leveraging on thyroid-specific targets derived from the Human Protein Atlas to measure tissue-specific RNA within the plasma was adopted. Genes that fall into the category of “Tissue-enriched genes” in thyroid tissues were selected (Uhlén et al., 2015; Atlas, 2021). Genes that are highly expressed and have more than four times fold-change when compared to other tissues were selected for analysis. Selected targets were also verified in literature to be biologically relevant before including into the panel (Lindahl et al., 2001; Afink et al., 2010; Li et al., 2012; Fernández et al., 2015; Soto-Pedre et al., 2017; Targovnik et al., 2017). Eventually, 11 thyroid-specific genes were targeted: TPO, TG, GFRA2, IYD, PDE8B, WDR86, C16orf89, DGKI, DIO2, TSHR, and PAX8.

### Circulating RNA Extraction

Consistent and reliable blood collection protocols are critical to maintain the integrity of circulating nucleic acid assays. We collected blood in Streck cfRNA tubes containing a stabilising reagent to prevent cell lysis and reduce degradation of RNA (Fernando et al., 2012).

cfRNA was extracted from 1 mL of plasma using Plasma/Serum Circulating and Exosomal RNA Purification Kit (Norgen, Cat no. 42800). The residual DNA in the cfRNA was digested using RNase-Free DNase I Kit (Norgen, Cat no. 25720). Extracted cfRNA was purified using RNA Clean and Concentrator^TM^-5 (Zymo, Cat no. ZYR.R1016), yielding 24 μL of cfRNA per sample.

### Reverse Transcription and Emulsion Based Targeted Pre-amplification

**FIGURE 1:**
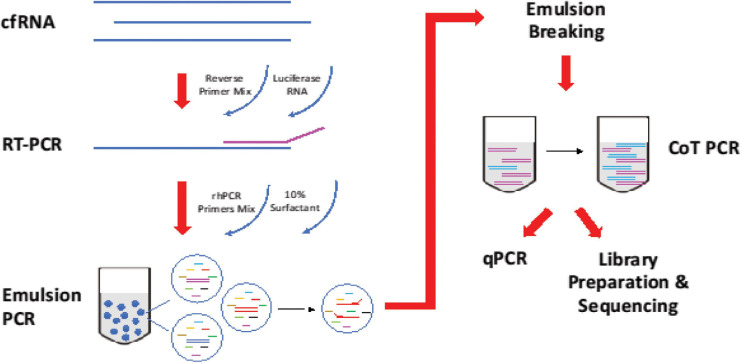
Workflow of reverse transcription and amplification of cfRNA. cfRNA, spiked-in with luciferase RNA control, was reverse transcript with Superscript^TM^ III Reverse Transcriptase (Invitrogen, Cat no. 18080044). The product was amplified with rhPCR primers and Platinum^TM^ Taq DNA Polymerase (Invitrogen, Cat no. 10966) using emulsion and CoT PCR. The residual primers were removed with Exonuclease I (New England Biolabs, Cat no. M0293). Amplified products were used for qPCR quantification and sequencing.

The multiplex approach is based on RNase H-dependent PCR (rhPCR), a method that provides increased target specificity over traditional PCR (Figure 1). Compared to traditional PCR, rhPCR requires an enzyme, RNase H2, and uses blocked primers (rhPrimers obtainable from IDT) in place of conventional PCR primers. rhPrimers contain a single RNA base and a 3′ blocking group. The blocking group must be removed by RNase H2 before extension by DNA polymerase. This additional condition results in less off target amplification and less primer-dimers are formed, resulting in increased specificity and efficient amplification of the circulating RNA that we are targeting. In this work, we specifically designed a panel of rhPrimers to amplify thyroid and housekeeping genes (Supplementary Table 1). In addition, we performed rhPCR in an emulsion that physically separated the reaction into multiple reaction-in-oil droplets, which aids in avoiding the formation of unproductive artefacts.

Sequencing is used primarily as quality control of the qPCR assay. This is to ensure that the amplicons match up to the intended primers design. Considerations were given to replace the qPCR assay with sequencing, however, due to the cost of sequencing which scale with each additional samples, we deployed it only once primarily to ensure that the amplicons generated are correct.

10 μL of extracted cfRNA was annealed with a final concentration of 0.4 μM of reverse primers mix in the presence of 10,000 copies of luciferase control RNA (Promega, Cat no. L4561) and a final concentration 2 mM of dNTPs at 65°C for 5 min. Reverse transcription of cfRNA was performed using Superscript^TM^ III Reverse Transcriptase (Invitrogen, Cat no. 18080044) at 25°C for 5 min, 50°C for 50 min, and enzyme inactivation at 95°C for 3 min.

cDNA from reverse transcription was added to the PCR mixture of Platinum^TM^ Taq DNA Polymerase (Invitrogen, Cat no. 10966) with a final concentration of 0.5 μM of rh PCR primers mix and 26 mU of RNase H2 enzyme. Emulsion was generated by adding three parts of 10% 008-FluoroSurfactant (RAN Biotechnologies) in 3M Fluorinert^TM^ Engineered Fluid (3M, Cat no. FC-40) to 1 part of PCR reaction mixture. The mixture was vortexed until it became cloudy and uniform. Thermocycling started with enzyme activation at 94°C for 2 min, followed by 20 cycles of denaturation (94°C, 15 s), annealing (55°C, 30 s), and extension (68°C, 1 min). Reaction recovered from emulsion PCR was top up with the same amount of polymerase and RNase H2 used in emulsion PCR. Thermocycling started with enzyme activation at 94°C for 2 min, followed by 20 cycles of denaturation (94°C, 15 s), hybridization (78°C, 10 min), annealing (55°C, 30 s), and extension (68°C, 1 min).

### Quantification of Pre-amplified Gene Targets Using qPCR

To monitor the expression of the targeted cfRNA across the different time point, qPCR was performed for 60 cycles with Maxima SYBR Green/ROX qPCR Master Mix (ThermoFisher Scientific, Cat no. K0221).

### Spike-In Controls and Quality Controls Assays for Ensuring Consistent Comparisons Across Patient Samples

There were two types of quality control. One for technical amplification efficiency and another for extraction efficiency.

Each sample is spiked with the same amount of commercially obtained Luciferase RNA that is not normally found in normal plasma (L4561, Promega). 10,000 copies of Luciferase Control RNA was spiked in with 10 μL of the extracted cfRNA at the reverse transcription step. The spiked-in served as a technical control for multiplex amplification efficiency control to normalise for any unintended variation in the experiment. Median *Ct* value of luciferase RNA across all samples is set as the benchmark. In each sample, all gene targets *Ct* values are offset till the Luciferase control *Ct* in the sample matches up to the benchmark.

Subsequently to normalise for possible variations during RNA extraction, geometric mean of the previous luciferase-adjusted *Ct* values for housekeeping genes (ACTB, GAPDH, and RPS18) are calculated and similarly offset in each sample till the housekeeping geometric mean matches up to the cohort average. In Supplementary Figure 1, we demonstrate that normalising using the luciferase and geometric mean of housekeeping genes result in tighter distribution of amplification curves.

### Data Analysis

Analysis of the data used normalised *Ct* values with respect to the housekeeping genes and corrected for amplification efficiency using spiked in luciferase RNA amount. All sequencing analysis was performed and plotted in R using ggplot. We started with normalising the raw *Ct* values with Luc and Housekeeping genes. We verified the amplified product by referring to the melt curve. For an undetermined *Ct*/incorrect product, *Ct* value of 60 was assigned. This was because 60 cycles were ran on qPCR. The final *Ct* value was then deducted from 60 to allow easy visualisation of the changes in the RNA expression.

Level of each circulating cfRNA was represented as median with interquartile range. Statistical significance was determined according to *p*-values generated by Wilcoxon Signed Rank *t* test. Stata software (Version15.1; StataCorp, TX, United States) was used for statistical analysis. The cfRNA level graphs were plotted using GraphPad Prism v9 (GraphPad Software).

## Results

### Multiplex Assay Is Capable of Amplifying and Quantifying Multiple Thyroid-Specific Genes in a Single Reaction

**FIGURE 2:**
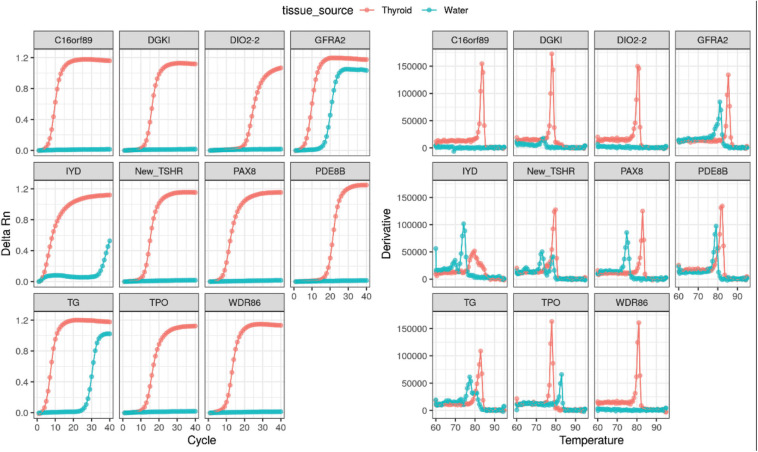
Positive amplification curves and melt curves of all thyroid-specific RNA transcripts using directly extracted thyroid RNA.

RNA extracted directly from the thyroid (commercially obtained from Takara Bioscience) was ran as a positive control using the assay. Successful amplification of these targets collaborates the notion that the selected genes were highly expressed within the thyroid tissue (Figure 2).

### Thyroid-Specific RNA Transcripts Are Present in Plasma and in Amounts Quantifiable Using Our Approach of qRT-PCR

The 11 selected thyroid-specific RNA transcripts (TPO, TG, GFRA2, IYD, PDE8B, WDR86, C16orf89, DGKI, DIO2, TSHR, and PAX8) were amplified from healthy volunteers (Supplementary Figure 2A) and thyroid patients (Supplementary Figure 2B). Both circulating DIO2-2 RNA and TSHR RNA were not detectable in healthy controls (Supplementary Figure 2A and Table 2). Similarly, in thyroid patients, only low level circulating DIO2-2 RNA was detected in 2 patients with *Ct* values of 56 and 58 (data not shown). The circulating TSHR RNA was only detected in a single time point of 7 patients (7/16, 44%) with *Ct* values ranging 36–58 (data not shown).

**TABLE 1:** Baseline characteristics of patients.

**Characteristics**	***n* = 27**
Age at diagnosis in years (median ± interquartile range)	54 (40–69)
**Gender, n (%)**	
Female	16 (59.3%)
**Ethnicity, n (%)**	
Chinese	14 (51.9%)
Malay	2 (7.4%)
Indian	1 (3.7%)
Others	10 (37.0%)
**Thyroglobulin Antibody Status**	
Present	17 (63.0%)
Absent	7 (25.9%)
Not known	3 (11.1%)
**Thyroid histology**	
Papillary thyroid cancer	22 (81.5%)
Follicular thyroid cancer	1 (3.7%)
Poorly differentiated thyroid cancer	1 (3.7%)
Benign thyroid nodule	3 (11.1%)
Including a patient with Grave’s disease	
And another with autoimmune thyroiditis	
**Thyroid cancer stage (AJCC eighth edition/TNM stage)**	
I	15 (55.6%)
II	5 (18.5%)
III	0
IVA	1 (3.7%)
IVB	3 (11.1%)
Not applicable for benign nodules	3 (11.1%)
**Risk of recurrence**	
Low	7 (25.9%)
Intermediate	7 (25.9%)
High	10 (37.1)
Not applicable for benign nodules	3 (11.1%)
**Response to therapy**	
Excellent	10 (37.0%)
Indeterminate	6 (22.3%)
Biochemical incomplete	3 (11.1%)
Structural incomplete	5 (18.5%)
Not applicable for benign nodules	3 (11.1%)
Follow-up duration (in months) (median ± interquartile range)	33 (21–46)

**TABLE 2:** Circulating levels of thyroid-specific cfRNA transcript candidates pre- and post-treatment in patients undergoing thyroidectomy or adjuvant radioactive iodine therapy.

**Total *n* = 27**	**Fall with treatment (surgery or RAI) (*n*)**	**Proportion of cohort with fall post-treatment**	**Mean level in healthy volunteers [60 - *Ct* value]**	**Median level pre-treatment of patients (interquartile range) [60 - *Ct* value]**	**Median level post-treatment of patients (interquartile range) [60 - *Ct* value]**	**% fall in median cfRNA level between pre- and post-treatment levels**	***p* value**
**TPO RNA**	**17**	**63%**	**27.4**	**19.8** **(2.7–21.3)**	**3.9** **(0–18.6)**	−80%	**0.0079**
**TG RNA**	**20**	**74%**	**18.9**	**32.1** **(29.4–33.6)**	**29.6** **(28.3 – 33.6)**	−8%	**0.0264**
**IYD RNA**	**20**	**74%**	**20.1**	**31.8** **(30.7–32.9)**	**30.3** **(28.3 – 32.9)**	−5%	**0.0058**
**GFRA2 RNA**	**19**	**70%**	**25.0**	**30.4** **(28.1–32.4)**	**28.2** **(26.5 – 29.1)**	−7%	**0.0110**
WDR86 RNA	4	15%	4.6	0 (0–0)	0 (0–6.3)	–	
PAX8 RNA	5	19%	10.3	0 (0–0.9)	1.9 (0–8.6)	–	
DGKI RNA	4	15%	8.0	0 (0–0)	0 (0–2.0)	–	
PDE8B RNA	5	19%	8.9	0 (0–0)	0 (0–0)	–	
C16orf89 RNA	4	15%	0	0 (0–0)	0 (0–0)	–	
TSHR RNA	2	7%	0	0 (0–0)	0 (0–0)	–	
DIO2-2 RNA	0	0%	0	0 (0–0)	0 (0–0)	–	

*Rows in bold represent cfRNA transcripts that fall post-treatment.*

### Thyroid-Specific cfRNA Levels Fall Following Surgical or Pharmacological Ablation of the Thyroid Tissue

**FIGURE 3:**
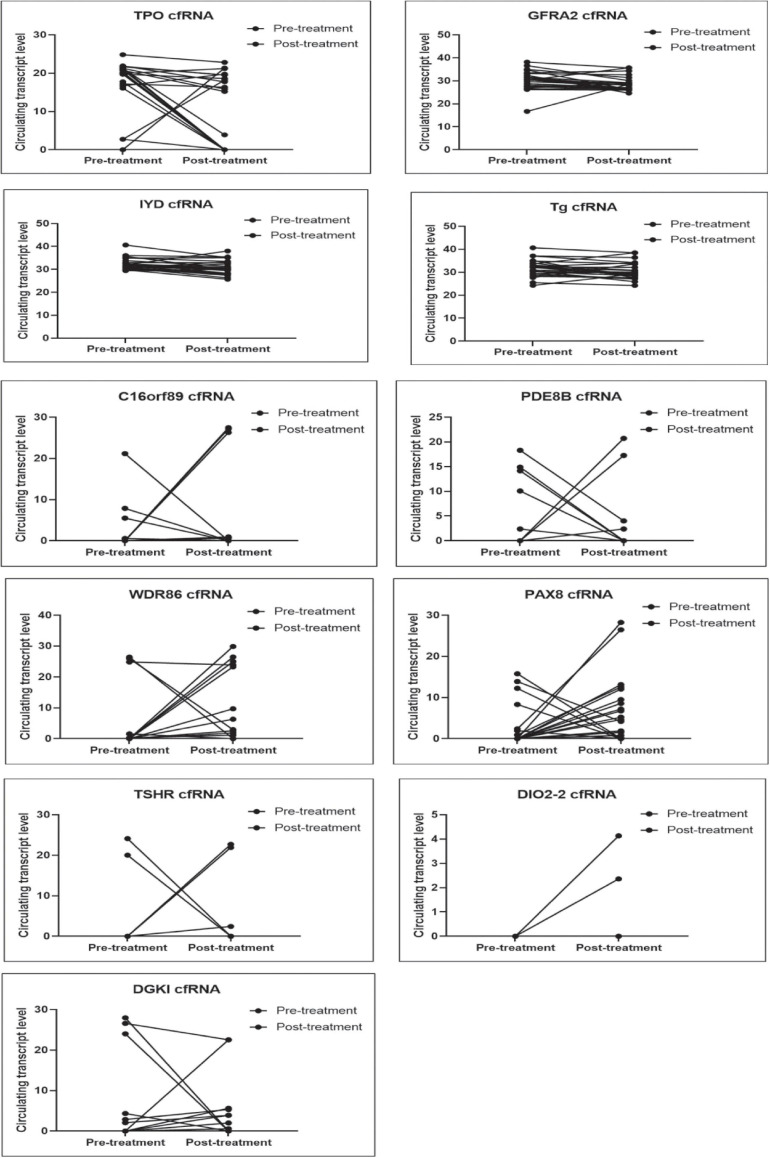
Plots comparing pre- and post- treatment levels of thyroid-specific cfRNA transcripts candidates. The circulating cfRNA levels plotted along Y-axis were derived from 60- *Ct* values.

We evaluated the 11 circulating thyroid-specific cfRNA levels in patients undergoing thyroid surgery for (i) benign thyroid conditions (e.g., benign thyroid nodules, hyperthyroidism from Graves’ disease; *n* = 3), (ii) or malignant thyroid nodules (*n* = 14), (iii) and patients with recurrent/persistent thyroid cancer undergoing repeat thyroid surgery or radioactive iodine adjuvant therapy (*n* = 10). All the thyroid cancer patients had papillary thyroid cancer (PTC) except for a patient with both PTC and poorly differentiated thyroid cancer, and another patient with follicular thyroid cancer. Their baseline characteristics is summarised in Table 1. Four of the circulating plasma cfRNA transcripts, namely TPO (thyroid peroxidase), IYD (iodotyrosine deiodinase), GFRA2 (glial cell line-derived neurotrophic factor family receptor alpha-2), and TG cfRNA, fell post-treatment in more than 50% of the study cohort, reaching statistical significance (Table 2 and Figure 3). The TPO cfRNA fell post-therapy in 63% of the study cohort with a magnitude of fall of 80%. Of note, in some patients, TPO cfRNA started to fall as early as 1 day post-treatment. The magnitude of fall in cfRNA level post-treatment in IYD, TG, and GFRA2 cfRNA were smaller at 5 to 8%. Similar to TPO cfRNA, in some patients, IYD, TG, and GFRA2 cfRNA started to fall as early as 1 day post-treatment supporting the potential for cfRNA to be early indicators of remnant thyroid tissue volume post-treatment.

### Thyroid-Specific cfRNA Transcripts Capture Temporal Trends in Clinical Course of Thyroid Cancer Patients

Temporal trends were showcased in three different clinical scenarios (peri-operative period, TG Ab positive state, recurrent/persistent thyroid cancer), reflecting different aspects where cfRNA measurements can make an impact.

**FIGURE 4:**
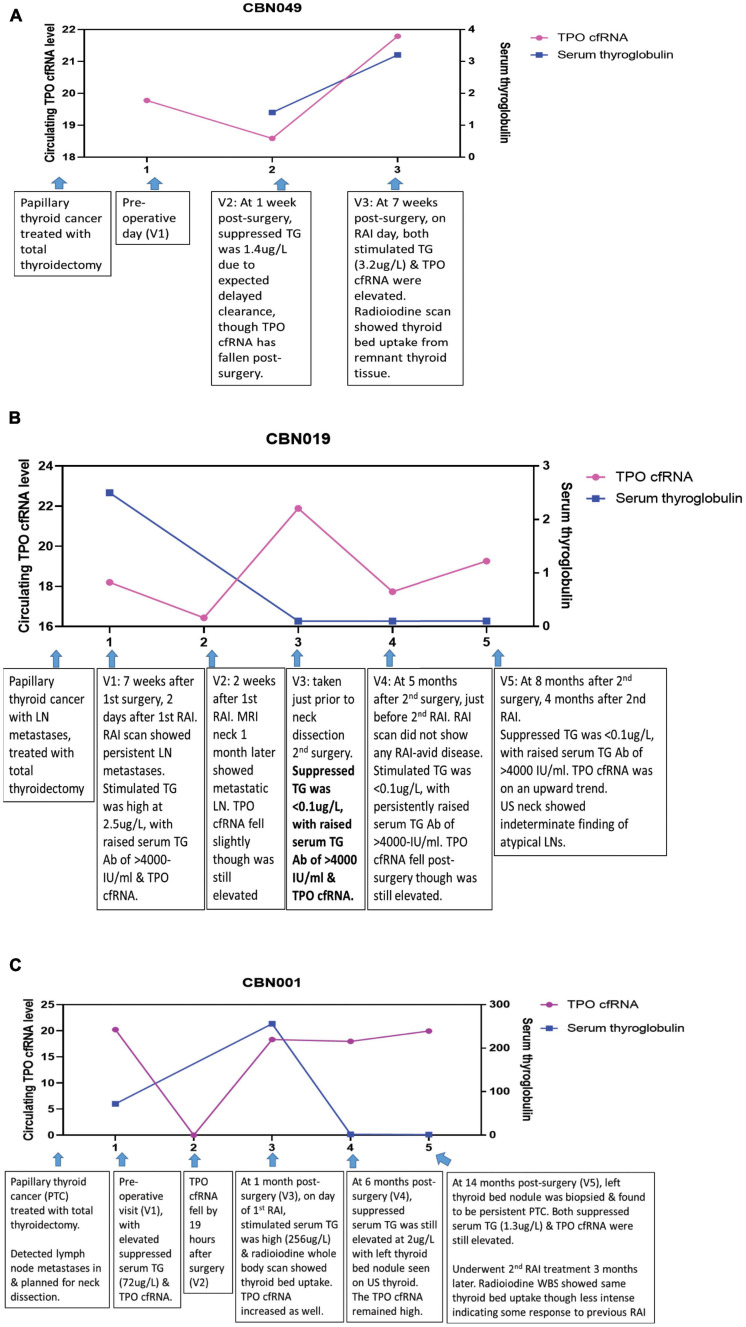
Longitudinal trend of thyroid-specific cfRNA in clinical course of thyroid cancer patients. The circulating cfRNA levels plotted along Y-axis were derived from 60- *Ct* values. **(A)** Peri-operative and pre-RAI TPO cfRNA level and serum thyroglobulin level at three time points in patient with papillary thyroid cancer. **(B)** TPO cfRNA level and serum thyroglobulin (TG) level in the setting of positive thyroglobulin antibody (TG Ab) in patient with papillary thyroid cancer and lymph node (LN) metastases. **(C)** TPO cfRNA level and serum thyroglobulin (TG) level in a patient with papillary thyroid cancer and persistent lymph node (LN) metastases undergoing further ablative therapy.

We demonstrated the expected variability of the observed TPO cfRNA level peri-operatively and under thyroid stimulating hormone (TSH) stimulation for adjuvant RAI therapy in a patient (CBN049) with Stage I papillary thyroid carcinoma [T3N0M0] (Figure 4A). Time point 1 was assessed pre-operatively, and time point 2 was measured 1 week post-operatively showing a fall in TPO cfRNA. The corresponding serum thyroglobulin level was 1.4 ug/L (not undetectable yet) as expected due to its delayed clearance. This patient did not have detectable TG Ab. As part of standard of care, in order for maximal RAI influx into remnant thyroid tissues for ablation, the TSH level is intentionally kept elevated either through thyroid hormone withdrawal or the administration of recombinant human TSH. Under this condition, thyroid tissues are also stimulated to produce maximal amount of thyroid proteins including thyroglobulin and TPO. This explains the elevated serum thyroglobulin and TPO cfRNA recorded at time point 3. This case illustrates that TPO cfRNA falls after thyroid surgery, and TPO transcription is increased by TSH stimulation as indicated by increased circulating cfRNA levels.

Next, we reviewed the utility of cfRNA measurement in a thyroid cancer patient with innate production of thyroglobulin antibodies (TG Ab) due to underlying autoimmune lymphocytic thyroiditis. In this clinical setting, TG Ab usually interferes with the serum thyroglobulin immunometric assay measurement. This patient (CBN019) had Stage I papillary thyroid carcinoma with cervical lymph node metastases [T2N1bM0], treated with total thyroidectomy and adjuvant RAI 7 weeks later (Figure 4B). At time point 1, evaluated 2 days after RAI under TSH stimulated state, serum TG was elevated at 2.5 ug/L, TG Ab was high at >4000 IU/ml (normal reference range <115 IUml), and TPO cfRNA was elevated as well. Her radioiodine scan showed persistent cervical lymph node metastases. At time point 2, measured 2 weeks later, her TPO cfRNA level fell slightly even though it was still elevated. MRI neck imaging performed 1 month after her adjuvant RAI therapy confirmed persistent cervical lymph node metastases. Time point 3 was taken just prior to neck dissection surgery for excision of the metastatic neck lymph nodes. By then her TPO cfRNA had risen though her serum TG was <0.1 ug/L, likely due to interference from the elevated TG Ab of >4000 IU/ml. At 5 months after her second surgery (time point 4), on the day of her second RAI in the TSH stimulated state, her TPO cfRNA fell even though it still remained elevated. Her serum TG was <0.1 ug/L with persistently elevated TG Ab. Her radioiodine scan did not show any RAI-avid disease. At time point 5, ascertained 4 months later, her TPO cfRNA level was on the upwards trend with similar undetectable serum TG and elevated TG Ab. Her neck ultrasound showed indeterminate finding of atypical lymph nodes that needed to be followed-up. This case illustrates the application of cfRNA technique for detection of persistent thyroid cancer in the setting of TG Ab positivity that interferes with TG immunometric assay.

We next analysed cfRNA time course for a patient (CBN001) who had persistent papillary thyroid cancer despite initial total thyroidectomy (Figure 4C). She had elevated serum TG post-surgery, in the absence of TG Ab, and was found to have cervical lymph node metastases. Just prior to neck dissection surgery, at time point 1, she had elevated serum TG (72 ug/L) and TPO cfRNA levels. After neck dissection surgery, her TPO cfRNA level fell within a day (time point 2). On day of her adjuvant RAI (time point 3), in the TSH stimulated state, both her serum TG (256 ug/L) and TPO cfRNA were elevated. Her radioiodine scan showed thyroid bed RAI uptake indicating residual thyroid tissue. At time point 4, during surveillance at 6 months after neck dissection, both serum TG (2 ug/L) and TPO cfRNA were still elevated, even though these levels were lower than levels detected at time point 1 (before neck dissection and RAI). Ultrasound scan of neck showed left thyroid bed nodule. This was later biopsied and proven to be persistent papillary thyroid cancer (time point 5). The biopsy needle washing TG level was elevated at 5180 ug/L, without detectable TG Ab. Concurrently, both serum TG (1.3 ug/L) and TPO cfRNA were elevated. Subsequently, she underwent another course RAI treatment. Her radioiodine scan showed the same thyroid bed RAI uptake though less intense, indicating that the previous RAI had partially ablated the tumour. This case illustrates that TPO cfRNA can be a potential tool to detect recurrent or persistent thyroid cancer, and its level can fall within a day after treatment, indicating that measurements are instant snapshots of the molecular physiology in time.

## Discussion

### Key Study Findings

In this study, we aimed to assess the performance of circulating thyroid-specific cfRNA in quantifying remnant thyroid gland tissue or malignancy. Circulating tissue-specific RNA had also been previously reported to be present at low levels. To increase sensitivity of our assay, we measured multiple thyroid-specific targets using a multiplex pre-amplification approach. In addition, a data-driven bioinformatics approach was adopted to design a multiplex primer design assay specific to thyroid targets with minimal cross-interaction. Integrating multiple measurements targeting the thyroid, we found the TPO cfRNA to be a potential circulating biomarker that can track the residual thyroid mass in patients, with levels falling dynamically as early as 1 day after treatment. We have demonstrated the clinical relevance of circulating TPO cfRNA by tracking the changes in levels throughout the patient clinical course in setting of the peri-treatment, recurrence, and TG Ab positive state.

The identification of a circulating cfRNA that changes in real-time and reflects molecular physiology in time, allows for further clinical study design to (1) examine the clinical utility in remnant thyroid mass quantification for precise guidance on RAI doses, and (2) to assess its performance in detecting thyroid cancer recurrence, especially in patients with TG Ab that interferes with existing serum TG immunometric assays.

### Harnessing Thyroid-Specific Targets Beyond Thyroglobulin

The current standard of care relies on quantification of circulating thyroid-specific protein TG to reflect thyroid tissue mass (Haugen et al., 2016). However, the most widely used TG assay, the immunometric assay underestimates serum TG in the presence of TG Ab due to the formation of TG-TG Ab complex that leads to a reduction of free TG, which is measured by immunometric assay (Rosario et al., 2021). Several studies had assessed the utility of TG measurement by LC-MS/MS. They had shown feasibility in detecting circulating TG via mass spectrometry technique. However, this needs to be further optimised to improve the current sensitivity of 40–60% as there were patients with structural disease and TG Ab with negative TG measured on LC-MS/MS (Spencer et al., 2014; Netzel et al., 2015; Azmat et al., 2016; Guastapaglia et al., 2020). TG measurement by radioimmunoassay (RIA) is an alternative technique for TG assessment in the presence of TG Ab though it had been reported to be associated with false-positive TG measurement in patients with TG Ab (Netzel et al., 2015). The assessment of circulating TG mRNA was shown to correctly identify 93% (13/14) of patients with structural disease, negative serum TG, in the presence of elevated TG Ab (Boldarine et al., 2010). This remains to be further validated.

We found that other circulating thyroid-specific targets such as TPO, IYD, TG, and GFRA2 cfRNA fell accordingly post-treatment for thyroid cancer, and could be potential biomarkers to reflect thyroid mass. Thyroglobulin (TG), thyroid peroxidase (TPO), and iodotyrosine deiodinase (IYD) are involved in thyroid hormone biosynthesis. Thyroid peroxidase oxidises iodide ions to iodine atoms for addition onto tyrosine residues on thyroglobulin, forming mono-iodotyrosine (MIT) and di-iodotyrosine (DIT). Thyroid hormones, thyroxine (T4) formation involves oxidative coupling of 2 DIT, whereas triiodothyronine (T3) *de novo* formation involves coupling of MIT and DIT. Some free MIT and DIT released by thyroid cells are scavenged by iodotyrosine deiodinase (also known as iodotyrosine dehalogenase 1, DEHAL1) to recycle iodide, preventing iodide from being leaked out of thyroid cells (Targovnik et al., 2017; Citterio et al., 2019). As such, circulating levels of TPO, IYD, and TG cfRNA whose function are specific to the thyroid gland could plausibly reflect thyroid mass. GFRA2 was found to be highly expressed in brain and thyroid tissue (Atlas, 2021), and it was enriched in papillary thyroid cancer from the TCGA dataset (Cancer Genome Atlas Research Network, 2014). It mediates activation of the RET tyrosine kinase receptor and is a candidate gene for RET-associated diseases. It had been shown to be present on immunohistochemistry stain in case series of papillary, follicular, and medullary thyroid cancer, as well as follicular adenoma (Lindahl et al., 2001).

The current tumour marker serum TG takes at least 4 weeks for complete clearance whereas levels of TPO cfRNA changes as early as 1 day after treatment. With further optimisation, it could potentially allowing for early quantification of residual thyroid mass and precise, timely planning of adjuvant RAI dosage after thyroid surgery.

### Limitations

The quantification provided in the form of 60-*ct* values for circulating cfRNA does not vary proportionally to the expected amount of thyroid mass. For example, the level of TPO cfRNA in patients with previous hemi-thyroidectomy is not half of the level in patients with an entire thyroid gland *in situ*. The cfRNA RT-qPCR technique requires further optimisation for better precision. Some patients with thyroid cancer did not shed TPO cfRNA into the circulation and had measurable circulating TG, GFRA2, IYD cfRNA levels instead. It is possible that different clonal thyroid cancer cells could preferentially produce different thyroid proteins due to genetic aberrations, and the use of a multiplex cfRNA panel that detects several thyroid-specific targets might be needed. The patient and healthy volunteer sample size is small. The authors have an ongoing study to recruit a larger cohort for validation of the findings in this study.

### Future Directions

Building on the foundation laid by the current study, future work could employ the digital emulsion PCR technique to quantify the thyroid-specific targets identified and interrogate if the sensitivity for detection of these low-abundance targets with small fold changes could be improved.

Conceptually, harnessing the presence of multiple circulating thyroid-specific cfRNA in the form of a multiplex thyroid transcript kit for the estimation of remnant thyroid tissue can potentially improve the sensitivity of detecting and quantifying thyroid mass; when complemented with the pre-existing tumour marker, serum TG that has high specificity, it has the potential of improving the accuracy of quantification of thyroid mass to guide RAI dosing decision and detect tumour recurrence even in the presence of TG Ab.

## Data Availability Statement

The original contributions presented in the study are included in the article/Supplementary Material, further inquiries can be directed to the corresponding authors.

## Ethics Statement

The studies involving human participants were reviewed and approved by NHG DSRB Study Reference Number: 2017/00632. The patients/participants provided their written informed consent to participate in this study. Written informed consent was obtained from the individual(s) for the publication of any potentially identifiable images or data included in this article.

## Author Contributions

SY, LK, and ET contributed to conception and design of the study. SY organized the clinical database. LK designed the primers. LK and KK performed the nucleic acid extraction and sequencing. SY and LK performed the statistical analysis, analysed the data, and wrote the first draft of the manuscript. SY, RP, KL, KN, WT, and TL contributed clinical samples. SY, LK, JN, RP, KN, and DN contributed to manuscript revision. All authors read and approved the submitted version.

## Conflict of Interest

The authors declare that the research was conducted in the absence of any commercial or financial relationships that could be construed as a potential conflict of interest.

## Publisher’s Note

All claims expressed in this article are solely those of the authors and do not necessarily represent those of their affiliated organizations, or those of the publisher, the editors and the reviewers. Any product that may be evaluated in this article, or claim that may be made by its manufacturer, is not guaranteed or endorsed by the publisher.
