# Supplementary material for: A Multiplex Thyroid-Specific Assay for Quantification of Circulating Thyroid Cell-Free RNA in Plasma of Thyroid Cancer Patients
Source: Front Genet. 2021 Aug 25;12:721832. doi: 10.3389/fgene.2021.721832 (PMC8425593; doi:10.3389/fgene.2021.721832)

**Supplementary Information**

**Table S1. Sequences of reverse primers, rh primers, and qPCR primers**

| **Category** | **Targets** | **Reverse Primers** | **rh Primers** | **qPCR Primers** |
| --- | --- | --- | --- | --- |
| Thyroid | TG Forward |  | GACGGAGCCAGAGATTTCCTrGTGATG/3SpC3/ | GACGGAGCCAGAGATTTCCT |
|  | TG Reverse | AAGCTGGTGGCCTTTGAGTT | AAGCTGGTGGCCTTTGAGTTrCCCCAC/3SpC3/ | AAGCTGGTGGCCTTTGAGTT |
|  | IYD Forward |  | TGAACACATCCCCTTCTCTCATrAACCAA/3SpC3/ | TGAACACATCCCCTTCTCTCAT |
|  | IYD Reverse | CCAGGGCTCTGTGTGA | CCAGGGCTCTGTGTGAGCrCCCACA/3SpC3/ | CCAGGGCTCTGTGTGA |
|  | GFRA2 Forward |  | GTGTGCCGGACTGACCACrCTGTGG/3SpC3/ | GTGTGCCGGACTGACCAC |
|  | GFRA2 Reverse | GACACGCCTGGTAATTGTCC | GACACGCCTGGTAATTGTCCrGCAGGC/3SpC3/ | GACACGCCTGGTAATTGTCC |
|  | TPO Forward |  | GCAGCAGAGATAATGGAAACrATCAAG/3SpC3/ | ACTCAACAATCACAGCATCC |
|  | TPO Reverse | AAGTGTTTGGGCATTTTG | AAGTGTTTGGGCATTTTGrGGGGCT/3SpC3/ | GCAGCATGTAAGGGAGAC |
|  | PAX8 Forward |  | TGGAGAAGATTGGGGACTArCAAACC/3SpC3/ | CATGTTTGCCTGGGAGAT |
|  | PAX8 Reverse | AGCTGTCCATAGGGAGGTT | AGCTGTCCATAGGGAGGTTrGAATGT/3SpC3/ | AATGGTTGCTGCACTTTG |
|  | PDE8B Forward |  | CTGGTTCCAAGTGATCGAArGCCAAT/3SpC3/ | ACCACTCTTCCAATGCCTAC |
|  | PDE8B Reverse | AGGAAAGAGTTGGTCCTTCC | AGGAAAGAGTTGGTCCTTCCrCGGGTC/3SpC3/ | CTCATCCAACTGATCGAGG |
|  | C16orf89 Forward |  | CTCTCAGACCTCTGCAGGArGCCTCT/3SpC3/ | GCTCTTCTTCCTCTGGGC |
|  | C16orf89 Reverse | TAGGGTAGGCGTATCCGAT | TAGGGTAGGCGTATCCGATrGGCCTG/3SpC3/ | CAGCTCTGCGGTTCAAGT |
|  | WDR86 Forward |  | ACTGCACCATCAGGAGGTrGGGACC/3SpC3/ | CAGTGTCTGCAGGTGTACC |
|  | WDR86 Reverse | AGCTGTCCGGTCATAGGA | AGCTGTCCGGTCATAGGArGCTGCC/3SpC3/ | GCTGCTGAAGAGCTGGTT |
|  | DGKI Forward |  | CCAGCTATGATTCGGATCTrCCCTGT/3SpC3/ | AGGAATCAGGCCAACATG |
|  | DGKI Reverse | AACTGATTTTGTTCACCCG | AACTGATTTTGTTCACCCGrGATCCC/3SpC3/ | GACGATCTGGGACAGACTG |
|  | DIO2 Forward |  | ACTCGGTCATTCTGCTCAArGCACGA/3SpC3/ | ATGCTGACCTCAGAGGGA |
|  | DIO2 Reverse | TCCATCTTTGCTAAAACCTG | TCCATCTTTGCTAAAACCTGrATGGAT/3SpC3/ | CCTGATGGAGGACAATTTAG |
|  | TSHR Forward |  | CTTACATGACGTCAATCCCTrGTGAAG/3SpC3/ | GTGAATGCTTTTCAGGGAC |
|  | TSHR Reverse | ACAGCATCCAGCTTTGTC | ACAGCATCCAGCTTTGTCrCCATTC/3SpC3/ | CCTTGGACTGAAGTAAAGCC |
| House-keeping | GAPDH Forward |  | ATGTTCGTCATGGGTGTGrAACCAG/3SpC3/ | AGAAGTATGACAACAGCCTCA |
|  | GAPDH Reverse | CATGAGTCCTTCCACGATA | CATGAGTCCTTCCACGATArCCAAAC/3SpC3/ | CCAAAGTTGTCATGGATGA |
|  | ACTB Forward |  | AGAAGAGCTACGAGCTGCCTGArCGGCCC/3SpC3/ | CATCACCATTGGCAATGA |
|  | ACTB Reverse | TGGAGTTGAAGGTAGTTTCG | TGGAGTTGAAGGTAGTTTCGrUGGATT/3SpC3/ | AAGGTAGTTTCGTGGATGC |
|  | RPS18 Forward |  | TATGCTCATGTGGTGTTGAGrGAAAGG/3SpC3/ | AGACATTGACCTCACCAAGA |
|  | RPS18 Reverse | AAGAACCAGTCTGGGATCTT | AAGAACCAGTCTGGGATCTTrGTACTC/3SpC3/ | TTGTACTGGCGTGGATTC |
| Control | LUC Forward |  | CTCTGATTAACGCCCAGCrGTTTTG/3SpC3/ | CTCTGATTAACGCCCAGC |
|  | LUC Reverse | GCGGTCGGTAAAGTTGTT | GCGGTCGGTAAAGTTGTTrCCATTA/3SpC3/ | GCGGTCGGTAAAGTTGTT |

**Figure S1. Normalization of Ct values using luciferase RNA spike ins and geometric means of housekeeping genes**


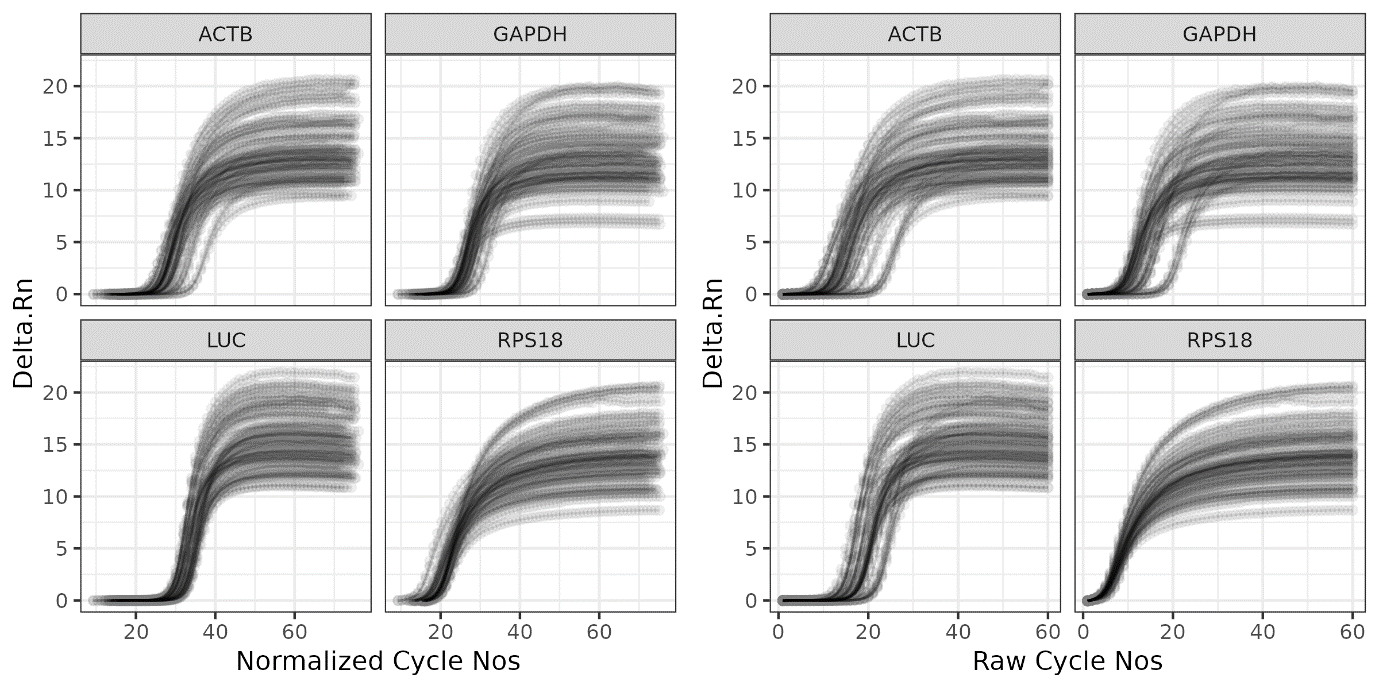


**Figure S2A. Ct values in amplification curve of 11 thyroid-specific RNA transcripts across clinical plasma samples from 2 health volunteers**

**
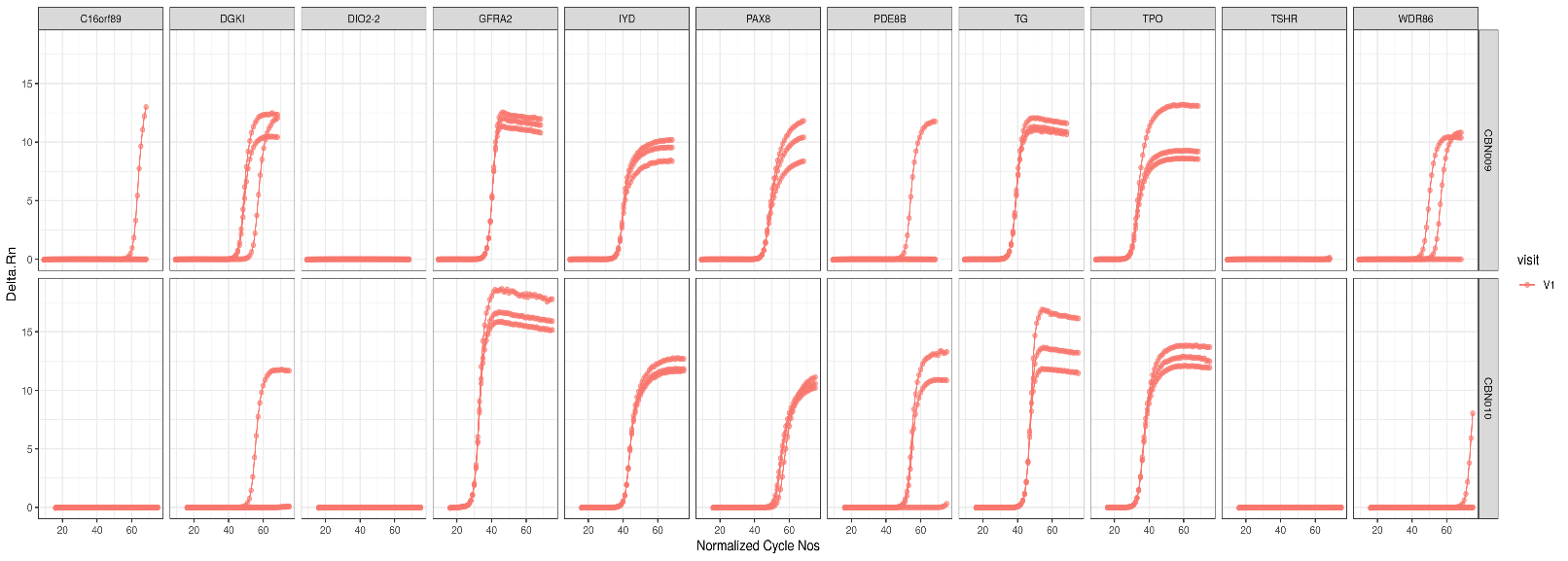
**

**Figure S2B. Ct values of TPO across clinical plasma samples of thyroid patients, Amplification curve & Melt curve of TPO**


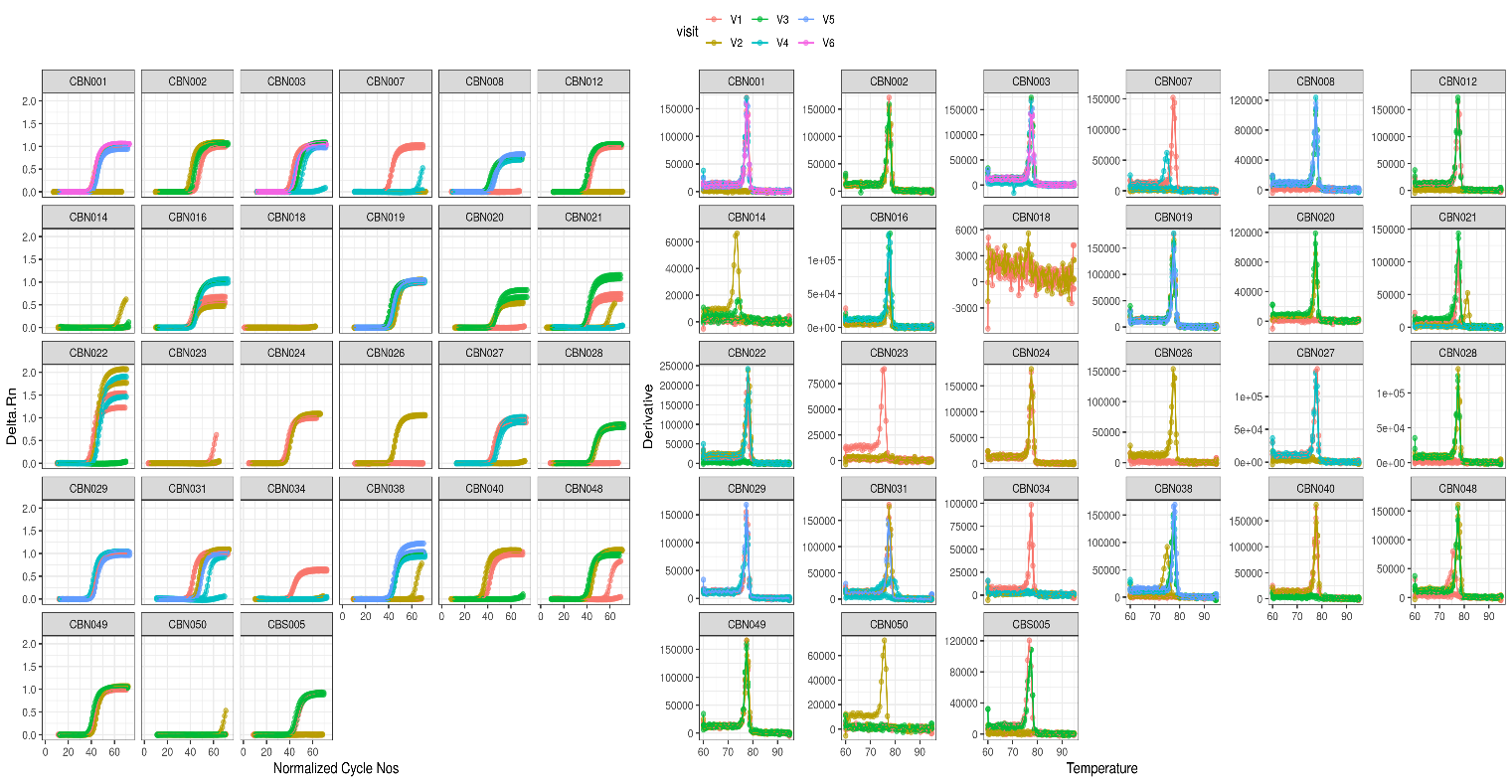

Supplement: Supplementary file 1 [file Data_Sheet_1.docx]
